# Plasma leakage in dengue: a systematic review of prospective observational studies

**DOI:** 10.1186/s12879-021-06793-2

**Published:** 2021-10-20

**Authors:** Chaturaka Rodrigo, Chathurani Sigera, Deepika Fernando, Senaka Rajapakse

**Affiliations:** 1 Department of Pathology, School of Medical Sciences, UNSW Sydney, Sydney, NSW 2052 Australia; 2 Kirby Institute, UNSW Sydney, Sydney, NSW 2052 Australia; 3 Department of Parasitology, Faculty of Medicine, University of Colombo, Colombo, 00800 Sri Lanka; 4 Department of Clinical Medicine, Faculty of Medicine, University of Colombo, Colombo, 00800 Sri Lanka

**Keywords:** Dengue, Plasma leakage, Critical phase, Haemorrhage, Systematic review

## Abstract

Plasma leakage is a precursor to life-threatening complications of dengue, but this group is poorly defined and not often reported in literature. Patients with Dengue haemorrhagic fever (DHF) as defined in the 1997 World Health Organization classification are often reported, and they all have plasma leakage, but some patients with plasma leakage do not meet the definition of DHF. The study aims to estimate the frequency of plasma leakage and DHF (as a surrogate of plasma leakage) in dengue and its variations based on virus serotype, geography, patient gender and pre-existing immunity to dengue. PUBMED, Scopus, EMBASE, CINAHL and Web of Science were searched for prospective observational studies reporting on plasma leakage or DHF. Quality of data was assessed using the NIH quality assessment tool for cohort studies. Forty-three studies that recruited 15,794 confirmed dengue patients were eligible. Cumulative frequency of plasma leakage was 36.8% (15 studies, 1642/4462, 95% CI 35.4–38.2%), but surprisingly the estimated cumulative frequency of DHF was higher (45.7%, 32 studies, 4758/10417, 95% CI 44.7–46.6%), indicating that current medical literature over-reports DHF or under-reports plasma leakage. Therefore, a reliable estimate for the proportion of dengue patients developing plasma leakage cannot be derived from existing medical literature even after applying rigorous inclusion criteria to select homogenous studies. Plasma leakage is an important marker of “at-risk” dengue patients and standardizing its definition, diagnosis and reporting should be a priority in research and global policy.

**Supplementary Information:**

The online version contains supplementary material available at 10.1186/s12879-021-06793-2.

## Background

An estimated 96 million symptomatic cases of dengue occur annually in 129 countries where the disease transmission is endemic [1, 2]. Burden of dengue is high in low–middle income countries where the health system comes under strain at times of seasonal epidemics [3, 4]. Nowadays, patient management and classification are standardised by guidelines published by the World Health Organization (WHO) or health departments in each country. The WHO guidelines published in 1997 classified the clinical spectrum of dengue as dengue fever and dengue haemorrhagic fever (DHF) with four different grades of DHF (I–IV) of increasing severity [5]. In a revised guideline published in 2009, this classification was changed as dengue fever and severe dengue [6]. Both classifications are currently used as one did not replace the other.

A typical case of dengue starts with a *febrile phase* characterised by fever, anorexia, headache, arthralgia, myalgia, or retro-orbital pain. Some patients progress to a subsequent *critical phase* characterised by increased capillary permeability and extravasation of fluid into interstitial space (plasma leakage), which occurs around day 5–7 of fever, and lasts for 48–72 h [5]. This is usually accompanied or preceded (by approximately 24–48 h) by a thrombocytopaenia. A progressive rise in haematocrit (evidence of haemoconcentration) or demonstration of extravasated fluid in pleural, peritoneal cavity by ultrasonography (or clinically) is used to establish the diagnosis of plasma leakage. Failure to recognise the onset of plasma leakage may lead to shock, multi-organ dysfunction syndrome and death. Alternatively, overenthusiastic fluid replacement during plasma leakage may lead to pulmonary oedema and its complications once this phase ends. Nevertheless, identification of this stage in illness is a critical for a favourable outcome. After the critical phase, patient enters a *recovery phase* characterised by resolution of symptoms and plasma leakage. Some patients move directly from febrile phase to recovery phase without a critical phase. As most complications in dengue stem from plasma leakage (except for a minority who can have abnormal bleeding and organ dysfunction without plasma leakage), identifying the proportion of patients progressing from *febrile phase* to *critical phase* is important for triaging at-risk patients for close monitoring either in an in- or outpatient basis, when all patients cannot be offered the same intense monitoring. Currently there is no reliable estimate for this proportion of patients, except for highly variable results reported from individual studies.

Both WHO classifications do not entirely capture the subgroup with plasma leakage, though the DHF category (in the 1997 classification) is a close approximation. Plasma leakage is an essential criterion to define DHF, but it is only one out of four such criteria (others being thrombocytopaenia, fever and haemorrhagic tendency) which must all be fulfilled to designate a patient as having DHF [5]. Therefore, all DHF patients have plasma leakage, but the converse is not true. While this classification is useful to identify patients before complications, waiting for all four criteria to be fulfilled leaves some people with plasma leakage at risk of being unattended. The 2009 WHO classification categories do not have a good overlap with the plasma leakage subgroup, though one criterion to define severe dengue in this classification is “severe plasma leakage”, while other criteria are “severe bleeding” and evidence of organ dysfunction. However, from a clinical point of view this is problematic as the options for clinicians are limited once these complications develop. Identifying all with plasma leakage is ideal to prevent complications, but this is ignored in this classification. Unfortunately, as most dengue cohorts report results according to WHO classifications, the proportion of patients developing plasma leakage (or critical phase in illness) is unknown. The objective of this systematic review is to fill this gap by estimating the proportion of patients developing plasma leakage in dengue from prospective observational studies, and to see variations of this estimate based on geographical origin, infecting serotype, gender, and pre-existing immunity against dengue.

## Methods

A systematic search was used to identify relevant studies published on or after 1997 (year of WHO publication that introduced the first major clinical classification on dengue), and indexed in PUBMED, Scopus, Web of Science, CINAHL and EMBASE databases using keywords “dengue” and (“plasma leakage” OR “critical phase” OR hemorrhag*), and (“prospective” OR “trial” OR “observational” OR “cohort”), without language restrictions. The last date of search was 15th April 2021. Full search strategy and the number of hits from each database is shown in Additional file 4: Table S1. Only prospective studies were included as for reliable reporting, the criteria for diagnosis of plasma leakage needs to be uniformly applied for all patients and this cannot be guaranteed in cross-sectional or retrospective study designs. Clinical trials were excluded as their interventions may influence the natural history of disease and case control studies were excluded as the sample size of patients with and without plasma leakage were pre-defined. Prospective observational cohorts with following characteristics were also deemed ineligible to preserve the quality of evidence; unconfirmed dengue diagnosis, reporting on ascites and pleural effusion separately (risk of counting same patient twice), sample size < 100 (arbitrary limit to exclude small cohorts to reduce variability), recruiting patients from some severity categories only (e.g., dengue shock syndrome) or exclusively from settings where a disproportionate number of severely ill patients are likely to be recruited (e.g. intensive care units). We included studies that recruited both children and adults, from any geographic region and regardless of whether recruitment was hospital or community based (but analysed separately). Anticipating that most eligible studies would not report on plasma leakage alone, an a priori decision was made to include DHF as a surrogate outcome when classification was done according to WHO 1997 criteria. Calculating DHF frequency is useful to validate the reliability of results as the percentage of patients with DHF must be lower than that for plasma leakage. Studies that used the WHO 2009 classification were included only if they reported the total number of patients with plasma leakage. Bibliography of included studies were manually searched for missed eligible studies.

**Fig. 1:**
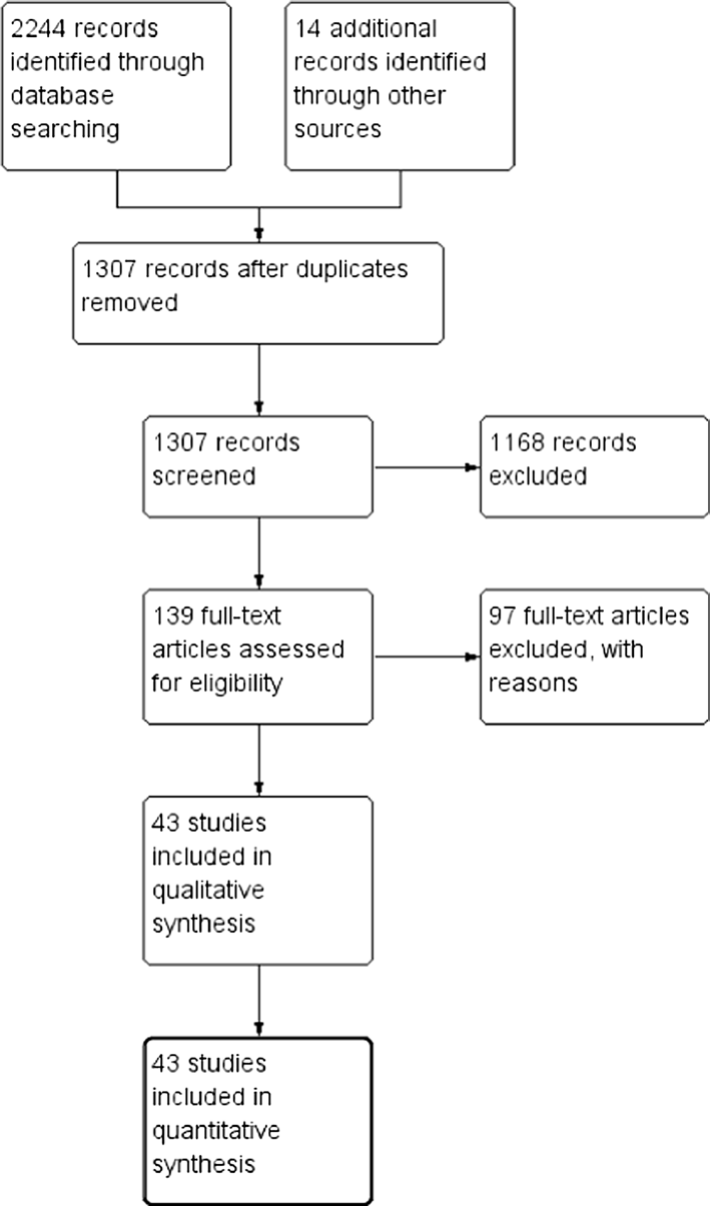
PRISMA flowchart of study selection

After removing duplicates in search results, all abstracts were independently screened by CR and CS and any disagreements were resolved by consensus of all authors. Full text articles were examined for all results identified during screening and conference abstracts were excluded (Fig. 1). From the final list of eligible studies, following data items were extracted; time window of recruitment, country of origin, method of dengue diagnosis, WHO classification system used in the study (or its variations), definition of plasma leakage, total sample size, total number of patients with plasma leakage (or DHF, if reported), number of deaths, distribution of plasma leakage or DHF in serotype, gender or pre-existing immunity-based subgroups (Tables 1, 2 and Additional file 4: Table S2). Only published data were considered. The quality of included studies were assessed with a modified NIH quality assessment tool [7] for observational cohort studies, and were categorized to a two tiered system with “Tier 1” having better quality evidence than “Tier 2” (Additional file 1). Summary estimates for proportion of patients with plasma leakage and DHF (and 95% confidence intervals) were calculated and comparisons were made across subgroups based on geographical origin, gender, infecting serotype, primary vs. secondary dengue infection and study quality (tier 1 studies vs. all included studies) to identify statistically significant differences, using Z test for proportions. A p value < 0.05 was considered as statistically significant. There is no published protocol for this review.

**Table 1:** Characteristics of included studies

Study	Recruitment period	Dengue diagnosis*	Community (C) or Hospital (H) based	Participants	Country	WHO classification system used	Reports DHF/PL/Both
Adil 2020 [8]	Sep 2019–Jan 2020	A, S	H	Adults	Pakistan	1997	Both
Alexander 2011 [9]	Aug 2006–May 2007	P, S	H**	Both	Thailand, Malaysia, Philippines, Nicaragua, Vietnam, Venezuela, Brazil	Both^#^	DHF
Avirutnan 2006 [11]	Nov 2001–Dec 2003	A, P	H	Children	Thailand	1997	DHF
Basuki 2010 [12]	Oct 2008–Apr 2009	P, S	H	Children	Indonesia	Both	Both
Biswas 2015 [13]	Aug 2005–Jan 2013	S, P, O	H**	Children	Nicaragua	Both	DHF
Bodinayaka 2018 [14]	Not mentioned	S, P, O	H	Both	Sri Lanka	2009	PL
Capeding 2015 [15]	Nov 2009–Nov 2010	S, P	H	Children	Philippines	1997	DHF
Chattergee 2017 [16]	Aug 2013–Jan 2014	A, S	H	Both	India	Both	DHF
Cordeiro 2007 [17]	Feb 2004–2006	A, S, P, O	H	Both	Brazil	1997	DHF
Fariz-Safhan 2014 [18]	Jul 2005–Jun 2006	S	H	Unclear	Malaysia	1997, 2002	Both
Guilarde 2008 [19]	Jan 2005–Jul 2005	S, P, O	H	Adults	Brazil	1997	DHF
Herath 2019 [20]	Aug 2016–Aug 2017	A	H	Adults	Sri Lanka	Not used	PL
Jagadishkumar 2012 [21]	Nov 2008–Jul 2010	S	H	Children	India	1997	DHF
Jain 2017 [22]	Aug 2015–Nov 2015	A, S	H	Adults	India	1997	DHF
Kirawittaya 2015 [23]	2010–2012	S, P	H	Children	Thailand	Both	DHF
Kittigul 2007 [24]	Sep 2003–Aug 2004	S	H	Both	Thailand	1997	DHF
Kularatnam 2019 [25]	July 2013–April 2014	S	H	Children	Sri Lanka	1997	DHF
Kulasinghe 2016 [26]	Apr 2013–Oct 2013	S	H	Children	Sri Lanka		PL***
Laul 2016 [27]	Jun 2015–Aug 2015	A, S	H	Adults	India	1997	DHF
Malavige 2006 [29]	Apr 2004–Jul 2004	S	H	Children	Sri Lanka	1997	DHF
Malavige 2006 [30]	Apr 2004–Jul 2004	S, P	H	Adults	Sri Lanka	1997	DHF
Manamperi 2019 [31]	Jan 2017–Dec 2017	A, S	H	Children	Sri Lanka	1997	DHF
Pham 2009 [32]	Jul 2007–Oct 2007	P, O	H	Adults	Vietnam	Not used	PL
Phuong 2004 [33]	Jun 1996–Jun 1998	S, O	H	Children	Vietnam	1997	DHF
Poeranto 2016 [34]^##^	1995–1999	S, P, O	H	Children	Indonesia	1997	DHF
Potts 2010 [47]	1994–2007	S, P, O	H	Children	Thailand	1997	DHF
Prasad 2020 [35]	Jul 2014–Jul 2015	S	H	Children	India	2009	PL
Premaratne 2013 [36]	Jul 2011–Dec 2011	S	H	Adults	Sri Lanka	1997	PL
Raman 2013 [37]	Feb 2011–Nov 2012	S	H	Adults	Bangladesh	1997, 2011	DHF
Seet 2006 [39]	Oct 2005–Nov 2005	S	H	Adults	Singapore	1997	DHF
Senaratne 2016 [40]	Jul 2011–Feb 2012	S, P	H	Adults	Sri Lanka	1997, 2011	DHF
Sigera 2021 [49]	Oct 2017–Feb 2020	A, P	H	Adults	Sri Lanka	2009	PL
Suwarto 2016 [41]	Mar 2010–Aug 2015	A, P	H	Adults	Indonesia	Not used	PL
Tang 2008 [48]	Aug 2006–Oct 2006	S	H	Both	China	1997	DHF
Taylor 2015 [42]	Sep 2008–Nov 2008	A, S, P	H	Adults	Vietnam	2009	PL
Thomas 2012 [43]	Jan 2005–Dec 2010	S, P	H	Adults	Martinique (France)	Both	Both
Trung 2012[50]	Sep 2006 – Sep 2008	A, S, P	H	Both	Vietnam	1997	PL^###^
Vasanwala 2014 [44]	Jan 2012–Aug 2012	A, P	H	Adults	Singapore	1997	DHF
Yacoub 2017 [45]	Jun 2013–Oct 2015	A, S, P	H	Both	Vietnam	2009	PL
Yung 2015 [46]	Apr 2005–Dec 2011	A, S, P	H	Adults	Singapore	Both	DHF
Anderson 2011 [10]	Jan 1998–2002	P, S	C	Children	Thailand	1997	DHF
L’Azou 2016 [28]	Jun 2011–Apr 2014	A, P	C	Children	Indonesia, Malaysia, Philippines, Thailand, Vietnam, Brazil, Honduras, Mexico, Puerto Rico, Colombia	1997	DHF
Sabchareon 2012 [38]	Feb 2006–2009	S, P, O	C	Children	Thailand	1997	DHF

*NS1 antigen test (A), RT-PCR (P), Serology—IgM or IgG and IgM combination or paired IgG analysis only (S), Other—viral isolation (O), **Mentions that some patients were managed as outpatients and proportion of in-patient DHF patients were extracted or calculated, for other studies all patients were assumed have been managed as in-patients, ***diagnosis of “DHF” was synonymous with plasma leakage, ^#^Primary study for 2009 WHO dengue classification, ^##^Reclassification of a previous cohort, ^###^This study provides the number of patients with a haematocrit rise > 20%, rather than plasma leakage

**Table 2:** Frequency of plasma leakage or dengue haemorrhagic fever (DHF) reported among laboratory confirmed dengue patients in included studies

Study	Plasma leakage			DHF		
	as % of symptomatic and asymptomatic patients (n/N)	as % of symptomatic patients (n/N)*	as % of symptomatic and hospitalised patients (n/N)**	as % of symptomatic and asymptomatic patients (n/N)	as % of symptomatic patients (n/N)*	as % of symptomatic and hospitalised patients (n/N)**
Adil 2020 [8]	–	–	38.33 (69/180)	–	–	41.11 (74/180)
Alexander 2011 [9]	–	–		–	50.12 (786/1568)	50.35 (715/1420)
Avirutnan 2006 [11]	–	–		–	–	69.94 (114/163)
Basuki 2010 [12]	–	–	27.59 (40/145)	–	–	51.72 (75/145)
Biswas 2015 [13]	–	–		–	24.97 (197/789)	28.68 (195/680)
Bodinayaka 2018 [14]	–	–	2.32 (9/388)	–	–	
Capeding 2015 [15]	–	–		–	–	83.67 (1496/1788)
Chattergee 2017 [16]	–	–		–	–	26.03 (57/209)
Cordeiro 2007 [17]	–	–		–	–	10.03 (29/289)
Fariz-Safhan 2014 [18]	–	–	62.5 (90/144)	–	–	59.72 (86/144)
Guilarde 2008 [19]	–	–		–	23.24 (43/185)	44.32 (39/88)
Herath 2019 [20]	–	–	37.43 (67/179)	–	–	
Jagadishkumar 2012 [21]	–	–		–	–	46.36 (51/110)
Jain 2017 [22]	–	–		–	–	46.34 (171/369)
Kirawittaya 2015 [23]	–	–		–	–	34.25 (62/181)
Kittigul 2007 [24]	–	–		–	–	94.76 (271/286)
Kularatnam 2019 [25]	–	–		–	–	23.08 (30/130)
Kulasinghe 2016 [26]	–	–	52.53 (83/158)	–	–	
Laul 2016 [27]	–	–		–	–	19.09 (21/110)
Malavige 2006 [29]	–	–		–	–	82.69 (86/104)
Malavige 2006 [30]	–	–		–	–	69.44 (75/108)
Manamperi 2019 [31]	–	–		–	–	48.41 (76/157)
Pham 2009 [32]	–	–	33.77 (51/151)	–	–	
Phuong 2004 [33]	–	–		–	–	50.55 (319/631)
Poeranto 2016 [34]^##^	–	–		–	–	21.36 (47/220)
Potts 2010 [47]	–	–		–	–	37.46 (236/630)
Prasad 2020 [35]	–	–	38.24 (39/102)	–	–	
Premaratne 2013 [36]	–	–	40.2 (41/102)	–	–	
Raman 2013 [37]	–	–		–	44.0 (88/200)	75.86 (88/116)
Seet 2006 [39]	–	–		–	–	19.69 (25/127)
Senaratne 2016 [40]	–	–		–	–	19.20 (43/224)
Sigera 2021 [49]	–	–	46.95 (200/426)	–	–	
Suwarto 2016 [41]	–	–	58.72 (101/172)	–	–	
Tang 2008 [48]	–	–		–	–	0 (0/353)
Taylor 2015 [42]	–	–	23.26 (30/129)	–	–	
Thomas 2012[43]	–	–	14.27 (102/715)	–	–	7.41 (53/715)
Trung 2012 [50]	–	–	46.43 (611/1316)	–	–	
Vasanwala 2014 [44]	–	–		–	–	20.24 (34/168)
Yacoub 2017 [45]	–	51.43 (126/245)	70.32 (109/155)	–	–	
Yung 2015 [46]	–	–		–	17.48 (82/469)	38.14 (82/215)
Anderson 2011 [10]	–	–		6.33 (36/569)	14.23 (36/253)	72.0 (36/50)
L’Azou 2016 [28]	–	–		–	4.24 (30/708)	28.85 (30/104)
Sabchareon 2012 [38]	–	–		–	10.66 (42/394)	21.76 (42/193)
Total	–	NC	36.79 (1642/4462)	NC	28.56 (1304/4566)	45.68 (4758/10417)

*Includes patients that were managed as in- or out-patients, **includes patients that were managed as in-patients only, *NC* Not calculated

## Results

Forty-three studies met the inclusion criteria [8–50] which included 40 hospital based and 3 community based cohorts that collectively recruited 15,794 patients (Table 1). Another ten studies [51–60] initially considered as eligible, were later excluded due to a high probability of reporting on same cohorts in the included studies. When two or more papers described the same cohort (e.g., interim analysis), the one that had a larger sample size was selected. Most included studies started recruitment after 1997, but three studies [33, 34, 60] had a recruitment time window straddling 1997, and one of these was a re-analysis of a prospective cohort that was active between 1995 and 1999. [34] Eighteen studies [10–13, 15, 21, 23, 25, 26, 28, 29, 31, 33–35, 38, 59, 60] included children only while others included both adults and children or adults only. The definition of children varied from those under 12 years (e.g., Sri Lanka) to less than 18 years of age (e.g., Philippines). Two studies [9, 28] were done in multiple countries while others reported from a single country and altogether 18 countries/territories were represented in included studies. Twenty-seven studies used the WHO 1997 criteria to classify patients, five used WHO 2009 criteria, and seven used both. One study [9] reported the primary data which formed the basis of the WHO 2009 clinical classification. Based on the quality assessment, seventeen studies were categorised as “Tier 2” studies, [12, 14, 16, 20, 22, 23, 26, 27, 29, 30, 35–37, 39, 42, 45, 48] and the rest as “Tier 1” studies (Additional file 2). Some studies reported on symptomatic hospitalised patients while others reported on all symptomatic patients regardless of hospitalisation, and one reported on asymptomatic patients in the community. These were analysed separately (Table 2) since hospitalised patients are more likely to be ill, have DHF or plasma leakage, and hence lumping all together in the analysis may create an artefact in the observed percentages. A comprehensive summary of characteristics of included studies (including the method of laboratory confirmation of dengue), is provided in Table 1. The PRISMA checklist for this review is provided as additional file 3.

### Plasma leakage

Fifteen studies reported the proportion of patients with plasma leakage out of all symptomatic and hospitalised patients (Table 2), but the method of diagnosis was inconsistent. Six studies predominantly relied on ultrasonography [8, 20, 32, 36, 41, 42] while the remainder used chest X-ray alone, [12] chest X-ray with ultrasonography, [35] ultrasonography and a rise in haematocrit > 20%, [43, 49] a rise in haematocrit (> 20% or > 15%) with clinical examination [18, 45, 50] or clinical examination alone [26]. One study did not mention the method of diagnosis [14]. With this variability, the cumulative percentage of plasma leakage in symptomatic, hospitalised dengue patients was 36.8% (1642/4462, 95% CI 35.4–38.2%) (Table 2). Only one study reported that some patients with plasma leakage were managed as outpatients, and in this study the percentage with plasma leakage was 51.4% out of all symptomatic patients (126/245), regardless of hospitalisation. None of the studies recruited non-hospitalised asymptomatic patients in the community so there is no data on the proportion of plasma leakage out of all dengue patients (symptomatic and asymptomatic).

The number of studies reporting on the breakdown of patients with plasma leakage across the subgroups of interest (gender, infecting serotypes, primary vs. secondary infection and geographical origin) were too few (n ≤ 3) for a meaningful analysis (Additional file 4: Table S2). Regarding the quality of studies, when only “Tier 1” studies were considered, the frequency of plasma leakage among symptomatic and hospitalised patients increased to 39.4% (1224/3104, 95% CI 37.7–41.2%) and this was a statistically significant increase (p < 0.0001).

### Dengue haemorrhagic fever

Thirty-two studies reported on the number of patients with DHF out of all symptomatic and hospitalised patients [Cumulative percentage: 45.7% (4758/10417), 95% CI 44.7–46.6%] (Table 2). Eight studies reported the number of DHF cases out of all symptomatic patients (whether hospitalised or not) and with this denominator, the cumulative percentage decreased to 28.6% (1304/4566, 95% CI 27.2–29.9%). One study [10] reported on both symptomatic and asymptomatic patients and in that study, the percentage with DHF was 6.3% of all dengue patients (36/569).

In the subgroup analysis (Additional file 4: Table S2), gender breakdown for DHF patients was available in 10 studies, breakdown by infecting serotype in 5–6 studies and breakdown as primary vs. secondary infection in 16 studies. DENV serotypes 2 or 3 infections were associated with a higher percentage of DHF compared to DENV 1 infection (p < 0.001). Secondary infection was more likely to result in DHF than in primary infection (p < 0.001). Regarding the geographical origin, cohorts from Asia (from South and Southeast Asia, n = 27) reported a significantly higher DHF percentage than those from Latin America and the Caribbean (collectively referred to as Americas, n = 5) (p < 0.001). Regarding the quality of studies, when only “Tier 1” studies were considered, the DHF percentage among hospitalised symptomatic patients increased to 47.7% (4098/8585, 95% CI 46.7–48.8%), and this was a statistically significant increment (p = 0.005).

### Deaths

Of all studies reporting on plasma leakage, six [8, 12, 14, 20, 32, 49] specifically mentioned no deaths, one (this study mentions a haematocrit rise > 20% rather than a “plasma leakage”) reported eight deaths [50], while the others did not confirm the presence or absence of deaths. Of all studies reporting on DHF, 26 reported on deaths (9 studies reported 60 deaths, [9, 15, 16, 18, 19, 21, 22, 30, 43] and 17 studies reported no deaths [8, 10–12, 14, 20, 23, 27–29, 32, 33, 37–39, 44, 49]). Thirty-five deaths occurred in DHF patients while for 24, it was not mentioned if they had DHF. Only one death was confirmed in a non-DHF patient. Overall, the dengue specific mortality was 0.6% (68/11,339).

## Discussion

This systematic review which assessed 43 prospective observational studies, that collectively recruited 15,794 laboratory confirmed dengue patients, could not resolve a reliable estimate for the number of patients developing plasma leakage in dengue. This unreliability stems from the observed higher frequency for DHF (compared to plasma leakage), as this cannot be true since patients with DHF are a subgroup of patients with plasma leakage. Either DHF is over-reported, or plasma leakage is under-reported, or both these scenarios may be true. The discussion will focus on few theories to explain this discrepancy. Also, almost all existing data refers to hospitalised symptomatic dengue patients with hardly any data on plasma leakage or DHF frequency among all dengue patients (symptomatic or asymptomatic, regardless of hospitalisation); This has a significant bias to inflate the proportion of patients with plasma leakage or DHF as hospitalised patients are closely monitored. There is also no data to compare the number of deaths across patient groups that had or did not have plasma leakage.

Plasma leakage is an important outcome in dengue to put on record as most complications occur within this group. Unlike plasma leakage, which is a biological phenomenon related to disease pathogenesis, [11, 61–65] other identifiers of “at-risk” patients such as shock may be confounded by medical mismanagement or patient driven factors such as oral fluid intake (in addition to disease pathogenesis). Definition and setting standards for diagnosing plasma leakage has been long neglected and as this review highlights, even after using rigorous inclusion criteria (prospective cohorts of confirmed dengue patients), the observed estimates for the proportion of patients with plasma leakage were highly variable across studies and led to an unreliable cumulative estimate when compared against the surrogate measure of DHF (which was more frequently reported, as it is part of the clinical classification in 1997 WHO guidelines).

The main reason for under-reporting plasma leakage is probably the lack of global standards to diagnose it. The studies we reviewed collectively used many methods to define plasma leakage, but each individual study mostly used only one of these methods. In this regard, recent studies mostly rely on ultrasonography while old studies relied on demonstrating a progressive increase of haematocrit from baseline. Some studies have relied on less sensitive methods such as clinical examination or chest radiographs to record this outcome. In our opinion, ultrasonography and haemoconcentration are both important to define plasma leakage while clinical examination or chest radiographs are unreliable and should not be used. Ultrasonography directly visualises fluid in third space, but interpretation is subject to operator expertise. Furthermore, at times of epidemics, the demand for scans is high and access is limited in resource limited settings. Haemoconcentration, as demonstrated by a 20% rise in haematocrit compared to baseline, is inexpensive and less time consuming, but establishing a baseline value may be difficult in a patient presenting late in illness. Given these reasons, there will always be a proportion of patients diagnosed by one of these methods but not the other, and if both methods are not used, the total number of patients will be under-reported. A working group comprising of public health experts and researchers in several countries recently attempted to define internationally acceptable endpoints in the clinical syndrome dengue, and the final consensus on what was defined as “moderate plasma leakage” is very similar to what is mentioned above except that a haematocrit cut-off of 15% is recommended, instead of 20% [66]. However, it may not be possible to capture asymptomatic patients or non-hospitalised patients with these outcomes as this will require evaluation in a clinical setting. A prospective cohort study conducted by us recently, out of 426 patients, 129 (30.3%) had plasma leakage confirmed by ultrasonography, 146 (34.8%) by a haematocrit rise > 20%, and 200 (46.7%) by either one of these methods [49]. While being positive by one method significantly increased the odds of being positive by the other, the non-overlap across the groups diagnosed by either method was also large (> 15% haematocrit rise may be a better cut-off). The final estimate for plasma leakage in this study is closer to and higher than the cumulative DHF frequency calculated in this systematic review and in our opinion, this is probably a closer observation to reality for symptomatic and hospitalised patients.

It is also possible that the frequency of DHF is overestimated in some of the included studies in this review. The local guidelines in some countries may not strictly adhere to the WHO criteria to define DHF (but still report it as DHF). For example, in our experience as clinicians in Sri Lanka, a diagnosis of DHF is synonymous with plasma leakage with less weighting given to other criteria such as thrombocytopaenia or bleeding. This will create a discrepancy in reporting against others who strictly rely on all four criteria to make designate a diagnosis of DHF. On top of that, the variation imposed by the diagnostic method for plasma leakage highlighted above, also affects the reported number of DHF patients.

Given these issues affecting the reliability of data, we have low confidence in the results observed for the subgroup analysis (Additional file 4: Table S2) and will not discuss it further. In our opinion, the chance of some of these results (associations with gender, infecting serotype and geographical origin) being outdated with more robust and reliable data reporting in future, are high. Having said that, the observed higher risk of DHF with secondary dengue infection is an expected finding given the current understanding of immunopathology of the disease [64]. There is also conflicting evidence that some serotypes cause more severe disease than others [67–71]. However, statistical power to compare multiple serotypes against disease severity is limited in a single centre study because typically a dengue epidemic is dominated by one serotype. To overcome this, long-term studies covering multiple epidemics in one place or meta-analyses as reported here will be needed, but with more robust data. Finally there is some anecdotal evidence that dengue in South and Southeast Asia leads to more adverse outcomes compared to the Americas as the age standardised incidence rates, age standardised death rates and disability adjusted life years (DALYs) lost, are higher in the former group of countries collectively, than in the latter [4]. However, this observation may also be influenced by a higher overall dengue incidence in Asia, discrepancies in access to healthcare and policy differences in each country affecting outcome definitions, clinical management and reporting (Additional files 2, 3).

Finally, due to variations in the definition of paediatric age group in different countries (12–18 years), it is not possible to estimate a cumulative plasma leakage (and DHF) frequency for adults and children from current medical literature using a systematic review as most studies will not agree with an arbitrary age limit used in a systematic review to define paediatric patients. However, data from individual studies suggest that DHF and plasma leakage frequencies may differ between adults and children. This is another issue which needs to be addressed by a global consensus.

This systematic review has several limitations and the main one as discussed above is the inability to reach a conclusion with current data. However, this is a limitation of the data rather than the method of the review. Regarding the methods, we did not further subgroup studies based on how they defined plasma leakage for two reasons; (a) not all studies mentioned this and (b) the number of studies in each subgroup will be few given the varying methods and their combinations used. We also removed studies with a sample size less than 100 with the intention of reducing variability introduced by small studies. This limit was arbitrary.

In conclusion, the proportion of dengue patients that have plasma leakage (or a critical phase in their illness) cannot be estimated from medical literature, which is a concern given how clinically important this subgroup is. This essentially prevents us from understanding how this outcome differs between adults and children, infections with different dengue serotypes, patient gender and geographical location. In dengue, patients at-risk must be identified before complications occur and defining this “at-risk” group as those with plasma leakage is a feasible, safer, and practical approach. This group will include most patients who would develop complications except for a minority with abnormal bleeding and organ dysfunction without plasma leakage. However, setting global standards to define this subgroup has long been neglected. We recommend that standardisation of diagnosis and reporting of plasma leakage in dengue should be a priority in research, and once a consensus is reached it should be applied globally. As a first-step, prospective dengue cohorts in future should report the number of patients with plasma leakage (and the method of diagnosis) in addition to the data routinely reported according to WHO clinical classifications.

## Supplementary Information

**Additional file 1. **Modified study quality assessment tool.

**Additional file 2. **Quality assessment of each included study.

**Additional file 3. **PRISMA checklist for a systematic review.

**Additional file 4: Table S1.** Search strategy and results. **Table S2.** Subgroup analysis of plasma leakage and dengue haemorrhagic fever (DHF) frequency.

## Data Availability

All data generated or analysed during this study are included in this published article and its Additional information files.

## Abbreviations

DHF: Dengue haemorrhagic fever
WHO: World Health Organization (WHO)
DALYs: Disability adjusted life years
