# Supplementary material for: Plasma leakage in dengue: a systematic review of prospective observational studies
Source: BMC Infect Dis. 2021 Oct 20;21:1082. doi: 10.1186/s12879-021-06793-2 (PMC8527656; doi:10.1186/s12879-021-06793-2)
Supplement: Supplementary file 1 — Additional file 1. Modified study quality assessment tool. [file 12879_2021_6793_MOESM1_ESM.docx]

| **Criteria** | **Yes** | **No** | **Unsure** |
| --- | --- | --- | --- |
| Study |  | | |
| 1. Was the research question or objective in this paper clearly stated? |  |  |  |
| 2. Was the study population clearly specified and defined? |  |  |  |
| 3. Was the participation rate of eligible persons at least 50%? |  |  |  |
| 4. Were inclusion and exclusion criteria for being in the study prespecified and applied uniformly to all participants? |  |  |  |
| 5. Was a sample size justification, power description, or variance and effect estimates provided? |  |  |  |
| 6. Was the diagnosis of dengue confirmed by a laboratory test?* |  |  |  |
| 7. Was the timeframe sufficient to detect an association between dengue diagnosis and DHF/ plasma leakage?* |  |  |  |
| 9. Were the same dengue diagnostic tests applied consistently across all study participants?* |  |  |  |
| 11. Were the outcome measures (DHF/ Plasma leakage) clearly defined, valid, and implemented consistently across all study participants? |  |  |  |
| 13. Was the attrition rate < 20%? |  |  |  |
| 14. Were outcomes reported by subgroups?* |  |  |  |
| Final grade |  | | |

**Supplementary material**

**Quality assessment tool of included studies**

*This table is a modified version of the quality assessment tool for observational cohort and cross-sectional studies available from National Heart, Lung and Blood Institute (NHLBI), USA*

***Modifications from the original NHLBI tool**

Some questions were modified to fit the purpose of this study while irrelevant questions from the original tool were dropped. The number against each question shows the corresponding question in the original NHLBI tool (the reader may see which questions were dropped and which ones were modified)

The original NHLBI tool is available at: <https://www.nhlbi.nih.gov/health-topics/study-quality-assessment-tools>

The quality was considered “Tier 2” if the combined total of “No” or “Unsure” answers were ≥ 3. The rest were categorized as “Tier 1”.
