# Supplementary material for: Plasma leakage in dengue: a systematic review of prospective observational studies
Source: BMC Infect Dis. 2021 Oct 20;21:1082. doi: 10.1186/s12879-021-06793-2 (PMC8527656; doi:10.1186/s12879-021-06793-2)
Supplement: Supplementary file 4 — Additional file 4: Table S1. Search strategy and results. Table S2. Subgroup analysis of plasma leakage and dengue haemorrhagic fever (DHF) frequency. [file 12879_2021_6793_MOESM4_ESM.docx]

**Supplementary Tables**

**Supplementary Table 1.** Search strategy and results (date of last search – 2021 / 04 / 15)*

| **Database** | **Search strategy** | **Number of hits** |
| --- | --- | --- |
| PUBMED | Dengue[Title/Abstract] AND ("plasma leakage"[Title/Abstract] OR "critical phase"[Title/Abstract] OR hemorrhag*)[Title/Abstract] AND (prospective[Title/Abstract] OR trial[Title/Abstract] OR observational[Title/Abstract] OR cohort)[Title/Abstract] | 559 |
| Scopus | TITLE-ABS-KEY ( dengue ) AND TITLE-ABS-KEY ( "plasma leakage" OR "critical phase" OR hemorrhag* ) AND TITLE-ABS-KEY ( prospective OR trial OR observational OR cohort ) | 812 |
| Web of Science | Search 1: TI = (dengue) AND TI = ("plasma leakage" OR "critical phase" OR hemorrhag*) AND TI = (prospective OR trial OR observational OR cohort)  Search 2: AB = (dengue) AND AB = ("plasma leakage" OR "critical phase" OR hemorrhag*) AND AB = (prospective OR trial OR observational OR cohort) | 315 (Search 1 + Search 2) |
| CINAHL | Search 1: TI = (dengue) AND TI = ("plasma leakage" OR "critical phase" OR hemorrhag*) AND TI = (prospective OR trial OR observational OR cohort)  Search 2: AB = (dengue) AND AB = ("plasma leakage" OR "critical phase" OR hemorrhag*) AND AB = (prospective OR trial OR observational OR cohort) | 65 (Search 1 + Search 2) |
| EMBASE | Search 1: TI = (dengue) AND TI = ("plasma leakage" OR "critical phase" OR hemorrhag*) AND TI = (prospective OR trial OR observational OR cohort)  Search 2: AB = (dengue) AND AB = ("plasma leakage" OR "critical phase" OR hemorrhag*) AND AB = (prospective OR trial OR observational OR cohort) | 424 (Search 1 + Search 2) |

*Based on reviewer’s advice the same search strategy was repeated including the word “vascular leakage” later for all databases, which found 91 results (without removing duplicates) and one more eligible study. The last date of search remains unchanged. The PRISMA diagram was updated appropriately.

**Supplementary Table 2.** Subgroup analysis of plasma leakage and dengue haemorrhagic fever (DHF) frequency

| **Subgroup** | **Plasma leakage** | | | **DHF** | | |
| --- | --- | --- | --- | --- | --- | --- |
|  | **Number of studies from which data is available** | **Cumulative % (n/N)** | **P value** | **Number of studies from which data is available** | **Cumulative % (n/N)** | **P value** |
| Gender |  |  |  |  |  |  |
| *Male* | **3** [1-3] | 31.5 (160/508) | - | **10** [4-13] | 25.55 (445/1742) | - |
| *Female* | **3** [1-3] | 17.74 (94/530) | <0.001 | **10** [4-13] | 28.55 (418/1464) | 0.056 |
| Serotype |  |  |  |  |  |  |
| *DENV1* | **1** [3] | 55.56 (25/45) | - | **6** [4, 9, 11, 14-16] | 12.62 (77/610) | - |
| *DENV2* | **2** [2, 3] | 24.76 (105/424) | <0.001 | **6** [4, 9, 11, 14-16] | 26.2 (164/626) | <0.001 |
| *DENV3* | **1** [3] | 63.83 (30/47) | 0.418 | **6** [4, 9, 11, 14-16] | 20.93 (148/707) | <0.001 |
| *DENV4* | **1** [3] | 66.67 (14/21) | 0.390 | **5** [4, 9, 11, 15, 16] | 8.33 (4/48) | 0.384 |
| Type of infection |  |  |  |  |  |  |
| *Primary* | **2** [2, 17] | 10.03 (31/309) | - | **16** [4-6, 8, 9, 11, 12, 14, 16, 18-24] | 14.09 (183/1299) | - |
| *Secondary* | **2** [2, 17] | 12.7 (80/630) | 0.234 | **16** [4-6, 8, 9, 11, 12, 14, 16, 18-24] | 38.27 (1117/2918) | <0.001 |
| Region^#^ |  |  |  |  |  |  |
| *Asia* | **13** [1, 3, 6, 17, 19, 25-32] | 38.21 (929/2431) | - | **27** [6-13, 15, 16, 18, 20-26, 33-41] | 51.75 (3717/7182) | - |
| *Americas* | **1** [2] | 14.27 (102/715) | NC | **5** [2, 4, 5, 14, 41] | 17.96 (326/1815) | <0.001 |

^#^Data from Alexander et al.[42] was excluded as the country specific breakdown of DHF cases was unavailable, ^#^This comparison is for hospitalised symptomatic patients only, while others are for all patients, NC – Not compared

**Supplementary table 3.** Definitions of plasma leakage and details of serological tests used to confirm primary vs. secondary dengue

| **Study** | **Plasma leakage definition** | **Dengue serology assessment technique** |
| --- | --- | --- |
| Adil 2020(1) | Ultrasonography and haemoconcentration  i.e.,>20% rise in hematocrit from the baseline, or presence of ascites, pleural effusion.  Variations: Abdominal ultrasound was done daily for all study participants on admission and during treatment, performed by two radiologists blinded to the clinical condition of the patient | Not mentioned |
| Alexander 2011(2) | As per the definition of DHF*.  Variations: Only patients with a minimum of 3 consecutive days of data, including at least one day within the critical period, were selected. Lowest haematocrit value between Day 3 and 9 of fever compared against the highest reading, and if all readings were high, the population mean was used. | Serology results are based on IgM and IgG Capture ELISA of paired specimens |
| Avirutnan 2006(3) | As per the definition of DHF*. | Not applicable |
| Basuki 2010(4) | Chest X-ray and clinical assessment  Frequency of monitoring not mentioned | Not mentioned  Quote: “*Blood sampling was performed for serological tests (IgG and IgM anti-dengue), PCR and culture and isolation of the virus*.” |
| Biswas 2015(5) | As per the definition of DHF*. | Quote: “*DENV-specific IgM antibodies observed by MAC-ELISA in paired acute- and convalescent phase samples; and/or a 4-fold increase in anti-DENV antibody titer measured using Inhibition ELISA in paired acute- and convalescent-phase samples*.” |
| Bodinayaka 2018(6) | Does not mention the method of diagnosis | IgG and IgM ELISA (further details not provided) |
| Capeding 2015(7) | As per the definition of DHF*. | Commercial IgM capture ELISA kit (PanBio; Alere, Brisbane, Australia). |
| Chattergee 2017(8) | As per the definition of DHF*. | Commercial ELISA kits – Dengue IgM capture ELISA (Inverness Medical Innovations, Cat No. E DEN01M/EDEN01M05) (MAC-ELISA) and Dengue IgG capture ELISA (Inverness Medical Innovations, Cat No. E-DEN02G) (GAC-ELISA). All ELISAs were performed on a fully automated EVOLIS platform. |
| Cordeiro 2007(9) | As per the definition of DHF*. | Commercial ELISA Kits - Anti-dengue IgM-capture ELISA (Bio-Manguinhos or PanBio, Brisbane, Australia) and Anti-dengue IgG indirect ELISA (PanBio) |
| Fariz-Safhan 2014(10) | As increase in haematocrit by at least 15%, or highest haematocrit level above 20% of age and gender adjusted population baseline, or >20% drop in haematocrit after fluid administration, or hypoalbuminaemia, supplemented with clinical examination | Commercial ELISA Kits - Dengue IgM Capture ELISA (Panbio, Australia, category no: E-DEN01M) and Dengue IgG Capture ELISA (category no: E-DEN02G) |
| Guilarde 2008(11) | As per the definition of DHF*. | In-house dengue MAC ELISA for both IgM and IgG |
| Herath 2019(12) | Ultrasonography  Quote: “*Ultrasonography was carried out a) when the platelet count dropped below 100,000/μl or b) when serial haematocrit monitoring showed haemoconcentration more than 20% of the baseline value or c) when there was clinical evidence of pleural effusion or ascites. If the initial scan did not show extravasated fluid, the scan was repeated every 8–12 h until fluid leakage was confirmed or the clinical condition improved*.” | Not applicable |
| Jagadishkumar 2012(13) | As per the definition of DHF*. | Dengue IgM capture ELISA (No further details) |
| Jain 2017(14) | As per the definition of DHF*. | IgM Capture ELISA, (National Institute of Virology, Pune, India) |
| Kirawittaya 2015(15) | Serial chest and abdominal ultrasonography and haemoconcentration as defined for DHF definition* | ELISA on paired sera samples (an assumption based on a reference given for serological testing) |
| Kittigul 2007(16) | As per the definition of DHF*.  Variations: an increase in haematocrit ≥20% above the average haematocrit for age and sex of the Thai population or the presence of pleural effusion or ascites. | Dengue Duo IgM and IgG Rapid Strip Test (PanBio, Brisbane, Australia) based on immunochromatographic assay. |
| Kularatnam 2019(17) | Ultrasonographic evidence of pleural or peritoneal fluid collection or progressive rise in haematocrit > 20%. | Commercial IgM capture ELISA (SD Diagnostics, Korea) |
| Kulasinghe 2016(18) | Clinical examination only | Not mentioned |
| Laul 2016(19) | As per the definition of DHF*. | Dengue IgM ELISA (NIV, Pune, India) |
| Malavige 2006(20) | As per the definition of DHF*. | Dengue Duo IgM and IgG Rapid Strip Test (PanBio, Brisbane, Australia) based on immunochromatographic assay. |
| Malavige 2006(21) | As per the definition of DHF*. | Dengue Duo IgM and IgG Rapid Strip Test (PanBio, Brisbane, Australia) based on immunochromatographic assay. |
| Manamperi 2019(22) | As per the definition of DHF*. | Not mentioned |
| Pham 2009(23) | Abdominal ultrasonography (done on day 5 of fever and repeated if necessary) | Not applicable |
| Phuong 2004(24) | As per the definition of DHF*.  Variations: For patients without a baseline haematocrit a cut-off of 44% was used. Population haematocrit value was considered as 37% (CXT Phuong, unpublished data), and a 20% increment of that would be 44%. | Paired serum samples were tested by ELISA (no further details provided) |
| Poeranto 2016(25) | As per the definition of DHF*.  Variations: Plasma leakage was also defined by fluid in the costophrenic angles in an upright chest radiograph. | Antibody levels were measured by plaque reduction neutralization tests (PRNTs) |
| Potts 2010(26) | As per the definition of DHF*.  Variations: Evidence of pleural effusion on a chest radiograph taken on the day after defervescence supplemented the haematocrit readings in deciding on plasma leakage | Dengue IgG and IgM ELISA (Further details not provided) |
| Prasad 2020(27) | Chest radiograph with ultrasonography | Commercial IgM capture ELISA (Panbio, Brisbane, Australia) |
| Premaratne 2013(28) | As per the definition of DHF*.  Variations: Used serial ultrasonography to identify plasma leakage | PanBio Dengue Duo-antibody Test (Brisbane, Australia) |
| Raman 2013(29) | As per the definition of DHF*. | Dengue IgM ELISA (no further details provided) |
| Seet 2006(30) | As per the definition of DHF*. | IgM and IgG antibodies to dengue using a double-sandwich capture ELISA (Innis et al., 1989 |
| Senaratne 2016(31) | As per the definition of DHF*. | Dengue IgM and IgG capture ELISA (Standard Diagnostics, Gyeonggi-do, Korea) |
| Sigera 2021(32) | Ultrasonography and a rise in haematocrit > 20%  First scan when the platelet count was <100,000/mm^3^, and then repeated 12 – 24 hours | Not applicable |
| Suwarto 2016(33) | Ultrasonography (frequency not mentioned) | Not applicable |
| Tang 2008(34) | WHO 1996 | Dengue IgM and IgG capture ELISA kits (Panbio, Brisbane, Queensland, Australia) were used to diagnose dengue infection according to the manufacturer’s protocol |
| Taylor 2015(35) | ultrasonography  ultrasound detected plasma leakage (pleural effusions/ascites) | dengue serology by IgM and IgG capture ELISA against DENV  Dengue serology. Serological samples were positive for IgM or IgG if the optical density (OD) units were 6 times higher than the negative control sera (i.e., units 12); all other results were classed as negative including “indeterminate” ratios of 8–< 12. A change from a negative to a positive result defined a seroconversion. |
| Thomas 2012(36) | ultrasonography and a rise in haematocrit > 20%  Plasma leakage was diagnosed if ultrasonography revealed ascites or pleural effusion or if hemoconcentration was demonstrated according to the measurement of hematocrit at admission. The hematocrit threshold for hemoconcentration was set at 45% in female patients and 47% in male patients, corresponding to a 20% increase of the normal levels recorded in Martinique | dengue-specific antibodies were detected by using immunoglobilin M (IgM) capture, immunoglobulin G (IgG) capture, and IgG indirect enzymelinked immunosorbent assay kits (Panbio, Brisbane, Australia). A positive IgG capture test result for a serum sample obtained within 6 days of fever onset indicated a secondary dengue infection. Serum samples with negative results by IgG capture and IgG enzyme-linked immunosorbent assay indicated a primary infection. |
| Trung 2012(37) | As per the definition of DHF*. | Dengue IgG and IgM capture ELISA on paired serum samples (further details not given) |
| Vasanwala 2014(38) | As per the definition of DHF*. | Not applicable |
| Yacoub 2017(39) | A rise in haematocrit by at least > 15% compared to baseline (or lowest haematocrit reading) when at least 3 haematocrit recordings were available during the acute illness. | Commercial IgG and IgM capture ELISA (Panbio, Australia) on acute and convalescent samples |
| Yung 2015(40) | As per the definition of DHF*. | Platelia™ NS1 ELISA (Bio-Rad Laboratories, Marnes-la-Coquette, France), PanbioÒ Dengue IgG Indirect, IgG Capture, and IgM Capture ELISAs (Alere Inc., Waltham, MA). |
| Anderson 2011(41) | As per the definition of DHF*. | IgG and IgM ELISA (further details not available) |
| L'Azou 2016(42) | As per the definition of DHF*. | Not applicable |
| Sabchareon 2012(43) | As per the definition of DHF*. | IgG and IgM ELISA (further details not available) |

**References**

1. Pham TB, Matheus S, Vu TQH, Deparis X, Marechal V: **Early clinical and biological features of severe clinical manifestations of dengue in Vietnamese adults**. *J Clin Virol* 2009, **45**(4):276-280.

2. Thomas L, Moravie V, Besnier F, Valentino R, Kaidomar S, Coquet LV, Najioullah F, Lengelle F, Cesaire R, Cabie A: **Clinical presentation of dengue among patients admitted to the adult emergency department of a tertiary care hospital in martinique: Implications for triage, management, and reporting**. *Annals of emergency medicine* 2012, **59**(1):42-50.

3. Suwarto S, Nainggolan L, Sinto R, Effendi B, Ibrahim E, Suryamin M, Sasmono RT: **Dengue score: A proposed diagnostic predictor for pleural effusion and/or ascites in adults with dengue infection**. *BMC infectious diseases* 2016, **16**(1).

4. Biswas HH, Gordon A, Nunez A, Perez MA, Balmaseda A, Harris E: **Lower Low-Density Lipoprotein Cholesterol Levels Are Associated with Severe Dengue Outcome**. *PLoS neglected tropical diseases* 2015, **9**(9):19.

5. Cordeiro MT, Silva AM, Brito CAA, Nascimento EJM, Magalhaes MCF, Guimaraes GF, Lucena-Silva N, de Carvalho EMF, Marques ETA: **Characterization of a dengiie patient cohort in Recife, Brazil**. *AM J TROP MED HYG* 2007, **77**(6):1128-1134.

6. Fariz-Safhan MN, Tee HP, Abu Dzarr GA, Sapari S, Lee YY: **Bleeding outcome during a dengue outbreak in 2005 in the East-coast region of Peninsular Malaysia: A prospective study**. *Tropical biomedicine* 2014, **31**(2):270-280.

7. Jain S, Mittal A, Sharma SK, Upadhyay AD, Pandey RM, Sinha S, Soneja M, Biswas A, Jadon RS, Kakade MB *et al*: **Predictors of dengue-related mortality and disease severity in a tertiary care center in north India**. *Open forum infectious diseases* 2017, **4**(2).

8. Potts JA, Gibbons RV, Rothman AL, Srikiatkhachorn A, Thomas SJ, Supradish PO, Lemon SC, Libraty DH, Green S, Kalayanarooj S: **Prediction of dengue disease severity among pediatric Thai patients using early clinical laboratory indicators**. *PLoS neglected tropical diseases* 2010, **4**(8).

9. Kirawittaya T, Yoon IK, Wichit S, Green S, Ennis FA, Gibbons RV, Thomas SJ, Rothman AL, Kalayanarooj S, Srikiatkhachorn A: **Evaluation of cardiac involvement in children with dengue by serial echocardiographic studies**. *PLoS neglected tropical diseases* 2015, **9**(7).

10. Kularatnam GAM, Jasinge E, Gunasena S, Samaranayake D, Senanayake MP, Wickramasinghe VP: **Evaluation of biochemical and haematological changes in dengue fever and dengue hemorrhagic fever in Sri Lankan children: A prospective follow up study**. *BMC pediatrics* 2019, **19**(1).

11. Poeranto S, Sutaryo S, Josef HK, Juffrie M: **A relationship between dengue virus serotype and the clinical severity in paediatric patients from Gondokusuman region, Yogyakarta between 1995 and 1999**. *Pediatr Med Rodzinna* 2016, **12**(3):318-325.

12. Tang Y, Kou Z, Tang X, Zhang F, Yao X, Liu S, Jin X: **Unique impacts of HBV co-infection on clinical and laboratory findings in a recent dengue outbreak in China**. *The American journal of tropical medicine and hygiene* 2008, **79**(2):154-158.

13. Vasanwala FF, Thein TL, Leo YS, Gan VC, Hao Y, Lee LK, Lye DC: **Predictive Value of Proteinuria in Adult Dengue Severity**. *PLoS neglected tropical diseases* 2014, **8**(2).

14. Guilarde AO, Turchi MD, Siqueira JB, Jr., Feres VC, Rocha B, Levi JE, Souza VA, Boas LS, Pannuti CS, Martelli CM: **Dengue and dengue hemorrhagic fever among adults: clinical outcomes related to viremia, serotypes, and antibody response**. *The Journal of infectious diseases* 2008, **197**(6):817-824.

15. Yung CF, Lee KS, Thein TL, Tan LK, Gan VC, Wong JGX, Lye DC, Ng LC, Leo YS: **Dengue serotype-specific differences in clinical manifestation, laboratory parameters and risk of severe disease in adults, Singapore**. *AM J TROP MED HYG* 2015, **92**(5):999-1005.

16. Sabchareon A, Sirivichayakul C, Limkittikul K, Chanthavanich P, Suvannadabba S, Jiwariyavej V, Dulyachai W, Pengsaa K, Margolis HS, Letson GW: **Dengue infection in children in Ratchaburi, Thailand: A cohort study. I. Epidemiology of symptomatic acute dengue infection in children, 2006-2009**. *PLoS neglected tropical diseases* 2012, **6**(7).

17. Bodinayake CK, Tillekeratne LG, Nagahawatte A, Devasiri V, Arachchi WK, Strouse JJ, Sessions OM, Kurukulasooriya R, Uehara A, Howe S *et al*: **Evaluation of the WHO 2009 classification for diagnosis of acute dengue in a large cohort of adults and children in Sri Lanka during a dengue-1 epidemic**. *PLoS neglected tropical diseases* 2018, **12**(2):15.

18. Avirutnan P, Punyadee N, Noisakran S, Komoltri C, Thiemmeca S, Auethavornanan K, Jairungsri A, Kanlaya R, Tangthawornchaikul N, Puttikhunt C *et al*: **Vascular leakage in severe dengue virus infections: A potential role for the nonstructural viral protein NS1 and complement**. *J INFECT DIS* 2006, **193**(8):1078-1088.

19. Kulasinghe S, Ediriweera R, Kumara P: **Association of abnormal coagulation tests with dengue virus infection and their significance as early predictors of fluid leakage and bleeding**. *SriLanka J Child Health* 2016, **45**(3):184-188.

20. Malavige GN, Ranatunga PK, Velathanthiri VGNS, Fernando S, Karunatilaka DH, Aaskov J, Seneviratne SL: **Patterns of disease in Sri Lankan dengue patients**. *Arch Dis Child* 2006, **91**(5):396-400.

21. Malavige GN, Velathanthiri VGNS, Wijewickrama ES, Fernando S, Jayaratne SD, Aaskov J, Seneviratne SL: **Patterns of disease among adults hospitalized with dengue infections**. *QJM Mon J Assoc Phys* 2006, **99**(5):299-305.

22. Manamperi M, Jayamanne BDW, Somaratne T, Perera N, Fernando L: **Predictive value of persistent NS1 antigen positivity beyond 3rd day for dengue haemorrhagic fever in Sri Lankan children**. *BMC research notes* 2019, **12**(1).

23. Phuong CX, Nhan NT, Kneen R, Thuy PT, van Thien C, Nga NT, Thuy TT, Solomon T, Stepniewska K, Wills B: **Clinical diagnosis and assessment of severity of confirmed dengue infections in Vietnamese children: is the world health organization classification system helpful?** *The American journal of tropical medicine and hygiene* 2004, **70**(2):172-179.

24. Senaratne T, Wimalaratne H, Alahakoon DGS, Gunawardane N, Carr J, Noordeen F: **Characterization of dengue virus infections in a sample of patients suggests unique clinical, immunological, and virological profiles that impact on the diagnosis of dengue and dengue hemorrhagic fever**. *Journal of medical virology* 2016, **88**(10):1703-1710.

25. Adil B, Rabbani A, Ahmed S, Arshad I, Sr., Khalid MA: **Gall Bladder Wall Thickening in Dengue Fever - Aid in Labelling Dengue Hemorrhagic Fever and a Marker of Severity**. *Cureus* 2020, **12**(11):e11331.

26. Basuki PS, Budiyanto, Puspitasari D, Husada D, Darmowandowo W, Ismoedijanto, Soegijanto S, Yamanaka A: **Application of revised dengue classification criteria as a severity marker of dengue viral infection in Indonesia**. *SOUTHEAST ASIAN J TROP MED PUBLIC HEALTH* 2010, **41**(5):1088-1094.

27. Herath H, Udeshika WAE, Samarawickrama SSM, Yogendranathan N, Jayamali WD, Kulatunga A, Rodrigo C: **Prediction of plasma leakage phase of dengue in resource limited settings**. *Clin Epidemiol Global Health* 2019, **7**(3):279-282.

28. Prasad D, Bhriguvanshi A: **Clinical Profile, Liver Dysfunction and Outcome of Dengue Infection in Children: A Prospective Observational Study**. *Pediatr Infect Dis J* 2020, **39**(2):97-101.

29. Premaratna R, Ragupathy A, Miththinda JK, de Silva HJ: **Timing, predictors, and progress of third space fluid accumulation during preliminary phase fluid resuscitation in adult patients with dengue**. *International journal of infectious diseases : IJID : official publication of the International Society for Infectious Diseases* 2013, **17**(7):e505-509.

30. Taylor WR, Fox A, Pham KT, Le HNM, Tran NTH, Tran GV, Nguyen BT, Nguyen MV, Nguyen LT, Yacoub S *et al*: **Dengue in adults admitted to a referral hospital in Hanoi, Vietnam**. *The American journal of tropical medicine and hygiene* 2015, **92**(6):1141-1149.

31. Yacoub S, Lam PK, Huynh TT, Ho HHN, Thi HTD, Van NT, Lien LT, Ha QNT, Le DHT, Mongkolspaya J *et al*: **Endothelial Nitric Oxide Pathways in the Pathophysiology of Dengue: A Prospective Observational Study**. *Clin Infect Dis* 2017, **65**(9):1453-1461.

32. Sigera PC, Weeratunga P, Deepika Fernando S, Lakshitha De Silva N, Rodrigo C, Rajapakse S: **Rational use of ultrasonography with triaging of patients to detect dengue plasma leakage in resource limited settings: a prospective cohort study**. *Tropical medicine & international health : TM & IH* 2021.

33. Capeding MR, L'Azou M, Manalaysay M, Vince-Woo CR, Rivera RG, Kristy Sy A, Mercado ES, Inobaya MT, Tayag EG: **Laboratory-confirmed Dengue in Children in Three Regional Hospitals in the Philippines in 2009-2010**. *The Pediatric infectious disease journal* 2015, **34**(11):1145-1151.

34. Chatterjee SS, Sharma A, Choudhury S, Chumber SK, Bage R, Parkhe N, Khanduri U: **Dengue fever in a south asian metropolis: A report on 219 cases**. *Iranian journal of microbiology* 2017, **9**(3):174-185.

35. Jagadishkumar K, Jain P, Manjunath VG, Umesh L: **Hepatic involvement in dengue fever in children**. *Iran J Pediatr* 2012, **22**(2):231-236.

36. Kittigul L, Pitakarnjanakul P, Sujirarat D, Siripanichgon K: **The differences of clinical manifestations and laboratory findings in children and adults with dengue virus infection**. *J Clin Virol* 2007, **39**(2):76-81.

37. Laul A, Laul P, Merugumala V, Pathak R, Miglani U, Saxena P: **Clinical Profiles of Dengue Infection during an Outbreak in Northern India**. *Journal of tropical medicine* 2016, **2016**:5917934.

38. Raman MH, Alam AYMS, Rahman AM, Khan MS, Shapla NR, Aleem MA: **Presentation, management and outcome of dengue fever - A study of 200 cases**. *J Med* 2013, **14**(1):18-22.

39. Seet RCS, Quek AML, Lim ECH: **Post-infectious fatigue syndrome in dengue infection**. *J Clin Virol* 2007, **38**(1):1-6.

40. Anderson KB, Gibbons RV, Thomas SJ, Rothman AL, Nisalak A, Berkelman RL, Libraty DH, Endy TP: **Preexisting Japanese encephalitis virus neutralizing antibodies and increased symptomatic dengue illness in a School-Based cohort in Thailand**. *PLoS neglected tropical diseases* 2011, **5**(10).

41. L'Azou M, Moureau A, Sarti E, Nealon J, Zambrano B, Wartel TA, Villar L, Capeding MR, Ochiai RL: **Symptomatic Dengue in Children in 10 Asian and Latin American Countries**. *The New England journal of medicine* 2016, **374**(12):1155-1166.

42. Alexander N, Balmaseda A, Coelho ICB, Dimaano E, Hien TT, Hung NT, Jäenisch T, Kroeger A, Lum LCS, Martinez E *et al*: **Multicentre prospective study on dengue classification in four South-east Asian and three Latin American countries**. *Trop Med Int Health* 2011, **16**(8):936-948.
